# Correction: Quantitative and Kinetic Proteomics Reveal ApoE Isoform-dependent Proteostasis Adaptations in Mouse Brain

**DOI:** 10.1371/journal.pcbi.1014764

**Published:** 2026-09-08

**Authors:** Nathan R. Zuniga, Noah E. Earls, Ariel E. A. Denos, Jared M. Elison, Benjamin S. Jones, Ethan G. Smith, Noah G. Moran, Katie L. Broce, Gerome M. Romero, Chad D. Hyer, Kimberly B. Wagstaff, Haifa M. Almughamsi, Mark K. Transtrum, John C. Price

Fig 3A and 3B are not displaying the data correctly. In addition, Figs 2–5 were supposed to be box plots instead of bar charts. Please see the correct Figs 2–5 here.

**Fig 2:**
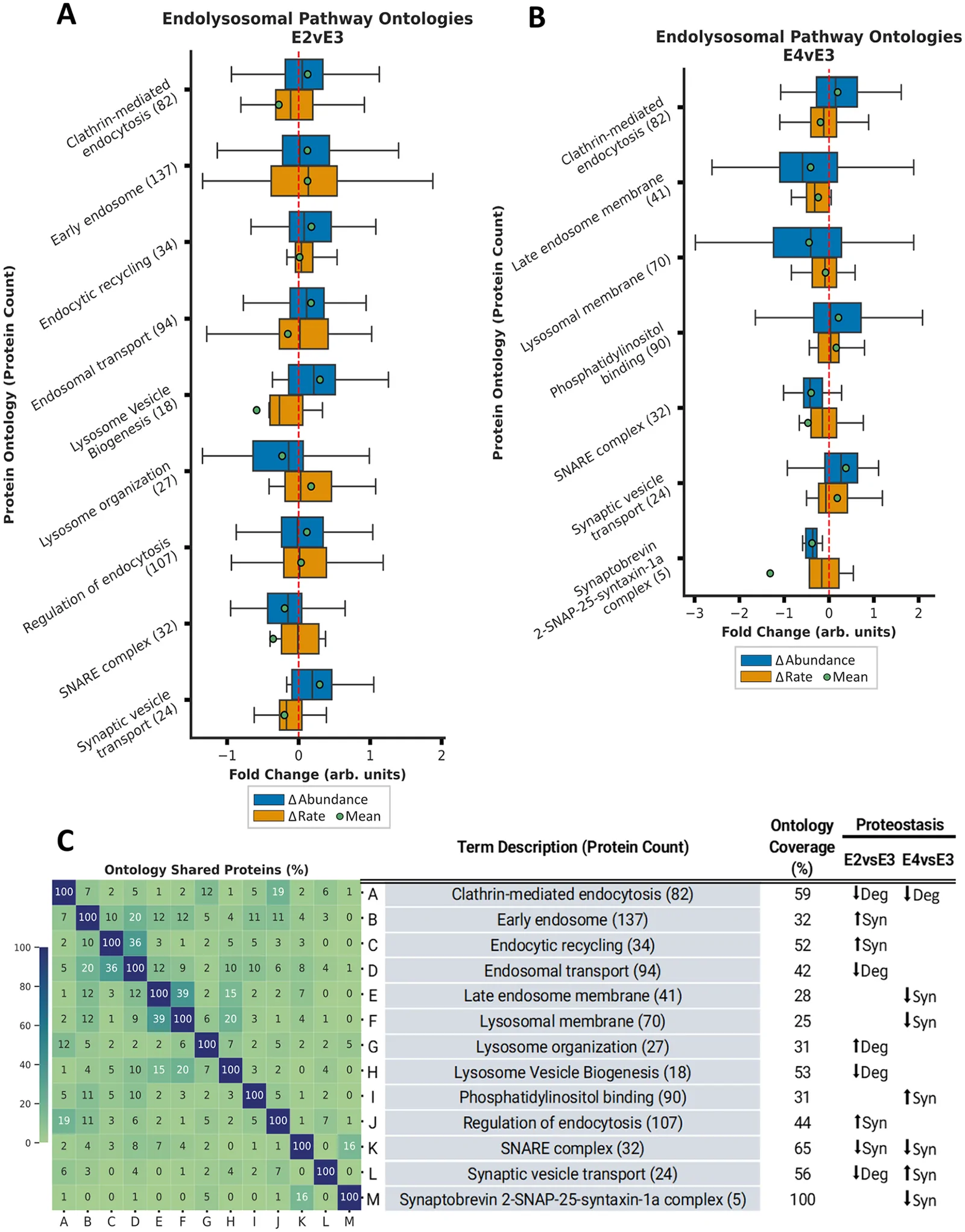
ApoE4 expression disrupts endosomal maturation and ApoE2 increases lysosomal capacity. **A-B.** Bar plot displaying Δabundance (orange) and Δturnover (blue) of proteins detected in all experimental cohorts for significant* ontologies related to endolysosomal trafficking in the E2vsE3 comparison (A) and in the E4vsE3 comparison (B). **C.** Heatmap displaying % of proteins shared across the endolysosomal ontologies with significant* Δabundance. (*BH-PV < 0.*05)*. Created with Matplotlib.py and InkScape.

**Fig 3:**
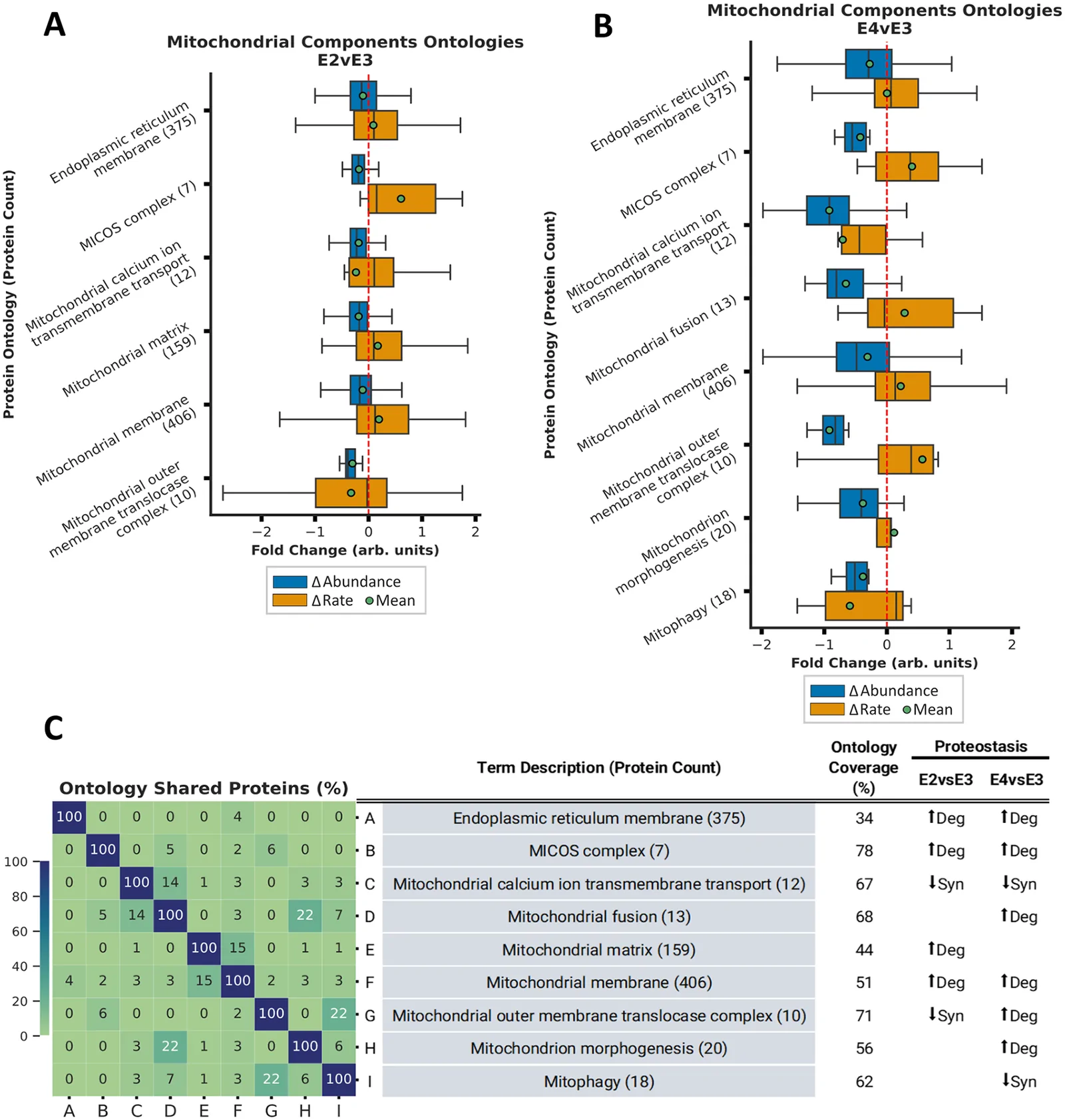
ApoE genotype differentially regulates mitochondrial proteostasis. **A-B.** Bar plot displaying Δabundance (orange) and Δturnover (blue) for ontologies of proteins detected in all experimental cohorts related to mitochondrial components with significant* Δabundance in the E2vsE3 comparison (A) and in the E4vsE3 comparison (B). **C.** Heatmap displaying % of proteins shared across the mitochondrial ontologies with significant* Δabundance. (*BH-PV < 0.*05)*. Created with Matplotlib.py and InkScape.

**Fig 4:**
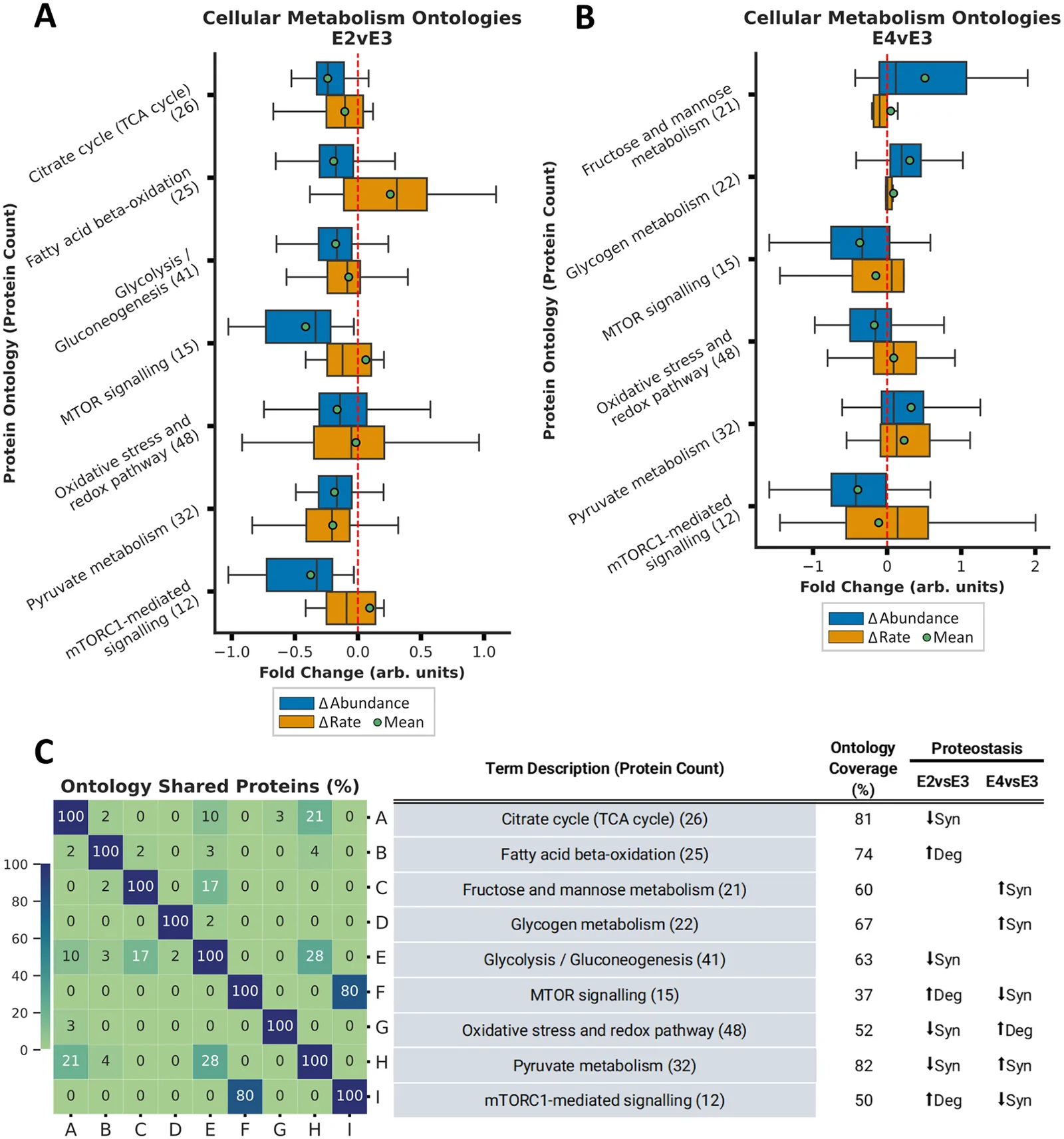
ApoE2 and ApoE4 expression drive changes in cellular fuel selection. **A-B.** Bar plot displaying Δabundance (orange) and Δturnover (blue) for ontologies of proteins detected in all experimental cohorts belonging to oxidative phosphorylation with significant* Δabundance in the E2vsE3 comparison (A) and in the E4vsE3 comparison (B). **C.** Heatmap displaying % of proteins shared across the oxidative phosphorylation ontologies with significant* Δabundance. (*BH-PV < 0.*05)*. Created with Matplotlib.py and InkScape.

**Fig 5:**
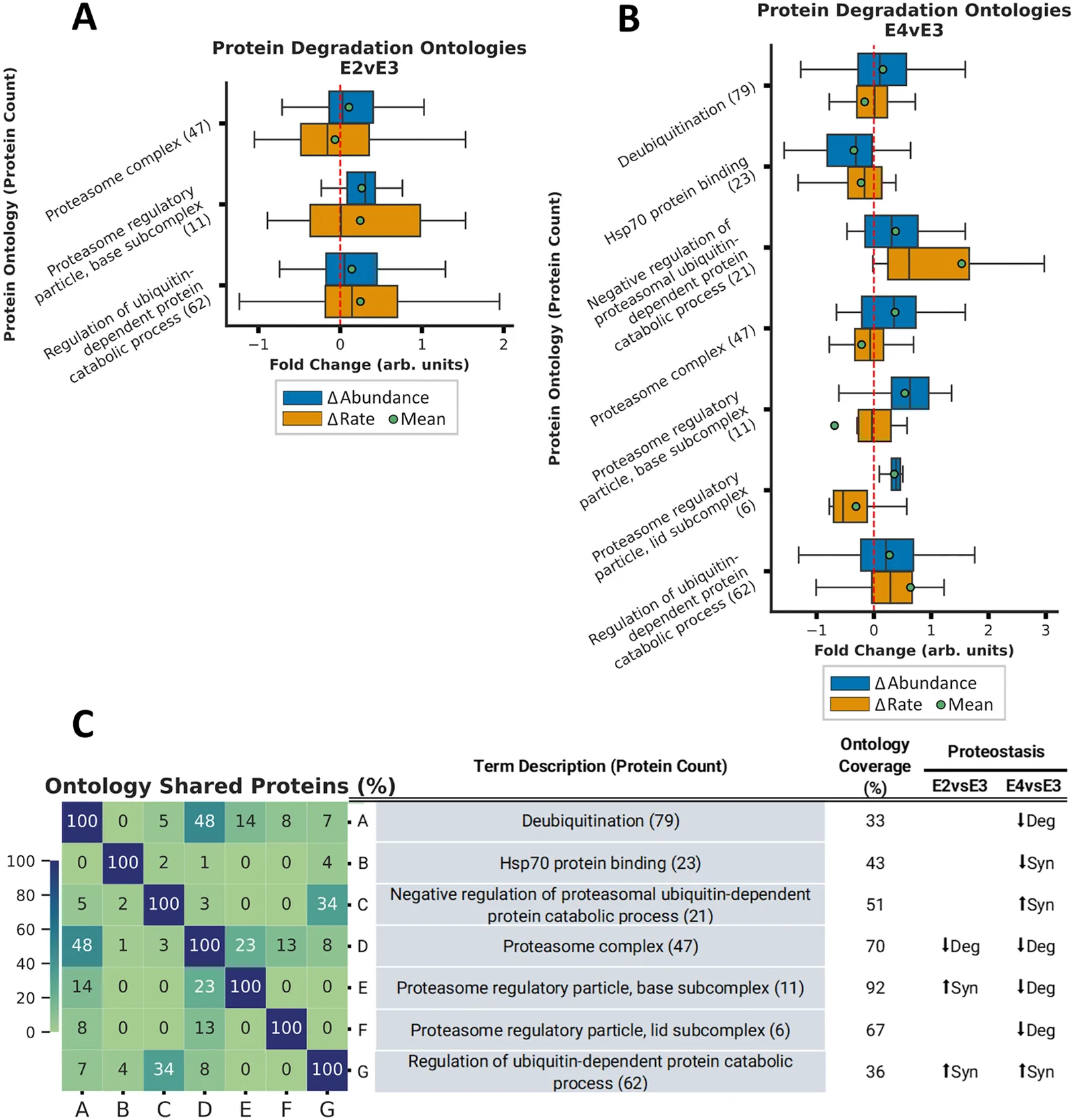
Proteasomal activity decreases with ApoE4 expression. **A-B.** Bar plot displaying Δabundance (orange) and Δturnover (blue) of proteins detected in all experimental cohorts for several ontologies related to proteasomal activity with significant* Δabundance in the E2vsE3 comparison (A) and in the E4vsE3 comparison (B). **C.** Heatmap displaying % of proteins shared across the ontologies with significant* Δabundance. (*BH-PV < 0.05). Created with Matplotlib.py and InkScape.
