# Supplementary material for: Quantitative and Kinetic Proteomics Reveal ApoE Isoform-dependent Proteostasis Adaptations in Mouse Brain
Source: PLoS Comput Biol. 2024 Dec 12;20(12):e1012407. doi: 10.1371/journal.pcbi.1012407 (PMC11671012; doi:10.1371/journal.pcbi.1012407)
Supplement: S1 File — Fig A. Detailed workflow chart describing both the mouse model and stages of analysis. Created with Biorender.com, Inkscape, Matplotlib.py, and Plotly.py. Fig B. Schematic of the minimum model for protein homeostasis applied to each protein Created with Inkscape, Matplotlib.py, and Plotly.py. Fig C. Abundance and turnover FCs for ontologies related to endolysosomal processes in A) E2vsE3 and B) E4vsE3 Created with Inkscape, Matplotlib.py, and Plotly.py. Fig D. Abundance and turnover FCs for ontologies related to mitochondrial components in A) E2vsE3 and B) E4vsE3 Created with Inkscape, Matplotlib.py, and Plotly.py. Fig E. Abundance and turnover FCs for ontologies related to cellular metabolism in A) E2vsE3 and B) E4vsE3 Created with Inkscape, Matplotlib.py, and Plotly.py. Fig F. Abundance and turnover FCs for ontologies related to protein degradation in A) E2vsE3 and B) E4vsE3 Created with Inkscape, Matplotlib.py, and Plotly.py. Fig G. Model for comparison of ApoE2-dependent changes between brain and liver. Created with Biorender.com and Inkscape. Fig H. Model for comparison of ApoE4-dependent changes between brain and liver. Created with Biorender.com and Inkscape. (DOCX) [file pcbi.1012407.s001.docx]

Supporting Information S1 File for: Quantitative and Kinetic Proteomics Reveal ApoE Isoform-dependent Proteostasis Adaptations in Mouse Brain

Nathan R. Zuniga^1^; Noah E. Earls^1^; Ariel E. A. Denos^1^; Benjamin S. Jones^1^; Chad D. Hyer^1^; Ethan G. Smith^1^; Noah G. Moran^1^; Katie L. Brown^1^; Jared M. Elison^1^; Kimberly Wagstaff^1^; Haifa M. Almughamsi^1,2^; Mark K. Transtrum^3^; John C. Price^*1^

^1^ Department of Chemistry and Biochemistry, College of Computational, Physical, and Mathematical Sciences, Brigham Young University, Provo, UT, USA

^2^ Department of Chemistry, College of Science, Taif University, Taif, Saudi Arabia

^3^ Department of Physics and Astronomy, College of Computational, Physical, and Mathematical Sciences, Brigham Young University, Provo, UT, USA

*Correspondence to [drjohncprice@gmail.com](mailto:drjohncprice@gmail.com)

*The box plots in the supplementary figures include additional ontologies that further support the findings of ApoE-related effects in the brains of transgenic mice. The boxplots also include outliers that were not present in the main text figures because of space limitations.*


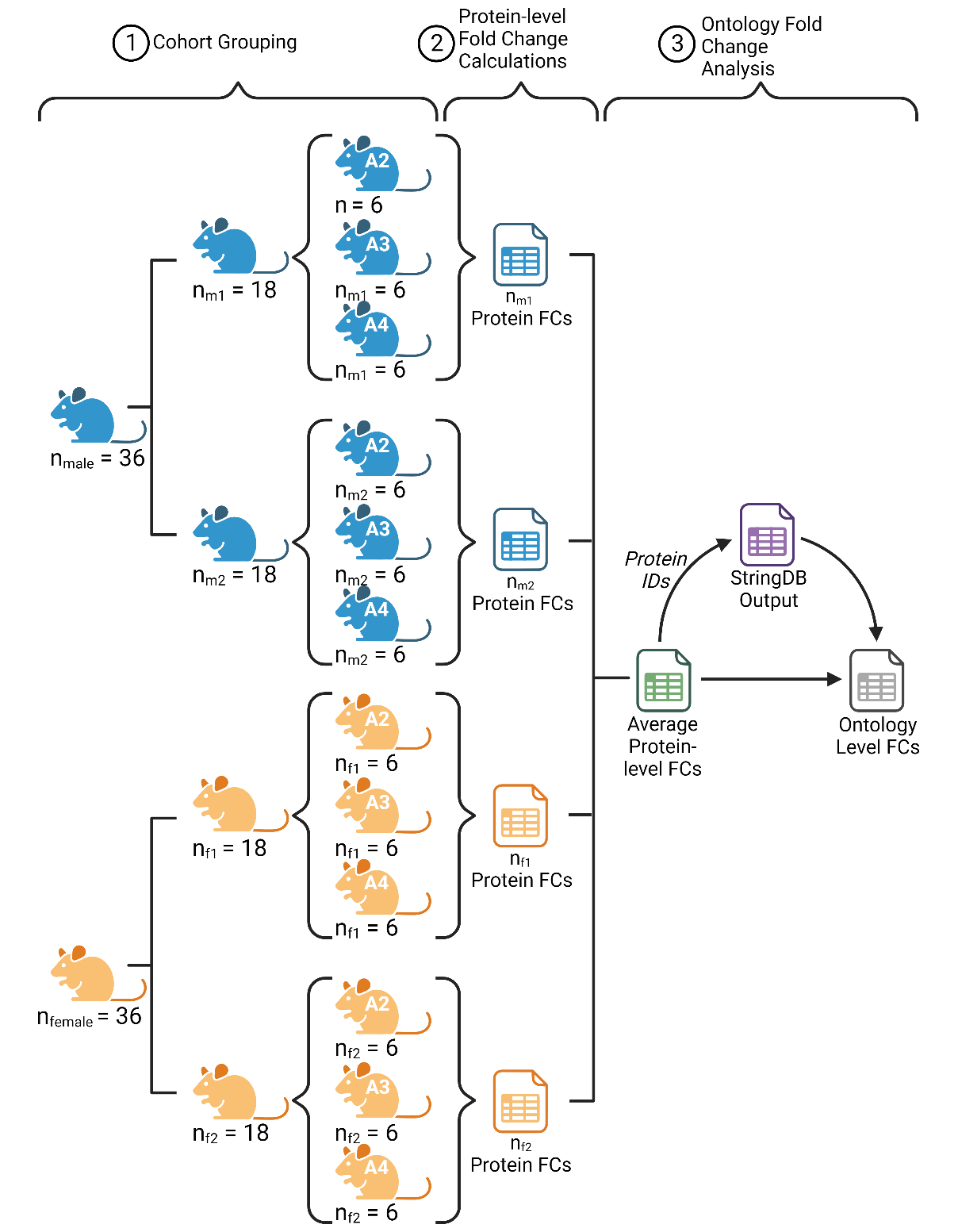


***Fig A. ApoE Cohort Design***

*72 mice were used to explore ApoE genotype. To facilitate sample preparation and accommodate for instrument availability, mice were split into 2 male groups and 2 female groups containing a total of 18 mice in each group. Each group consisted of 6 homozygous mice of each ApoE genotype. Data from each group was analyzed independently to calculate abundance and turnover fold changes. Proteins with a quantified abundance fold change were analyzed with StringDB multiprotein tool to identify ontologies represented in those proteins. Finally, ontology level fold changes were calculated using protein fold changes from abundance and turnover data.*

***
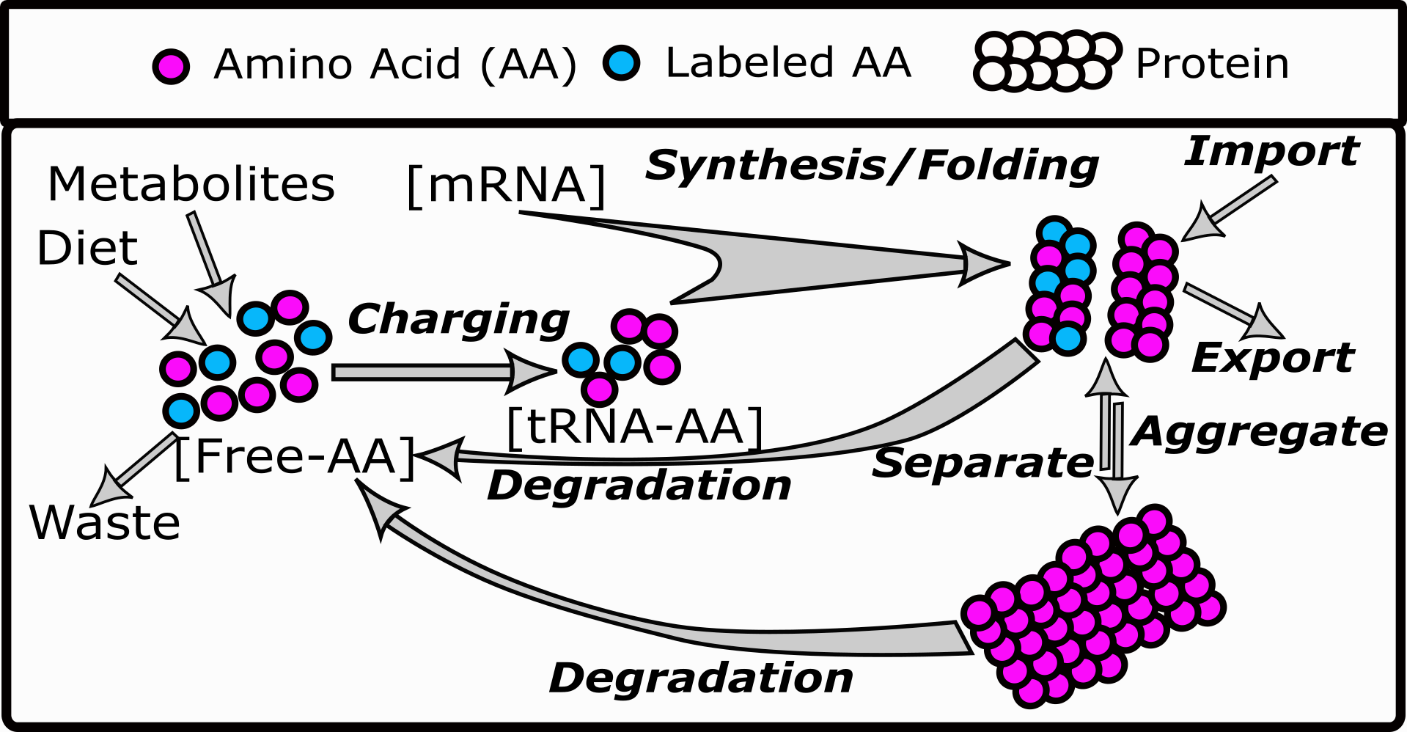
***

**Fig B.** *Protein Homeostasis Model with commonly observed sources and sinks of protein*

 
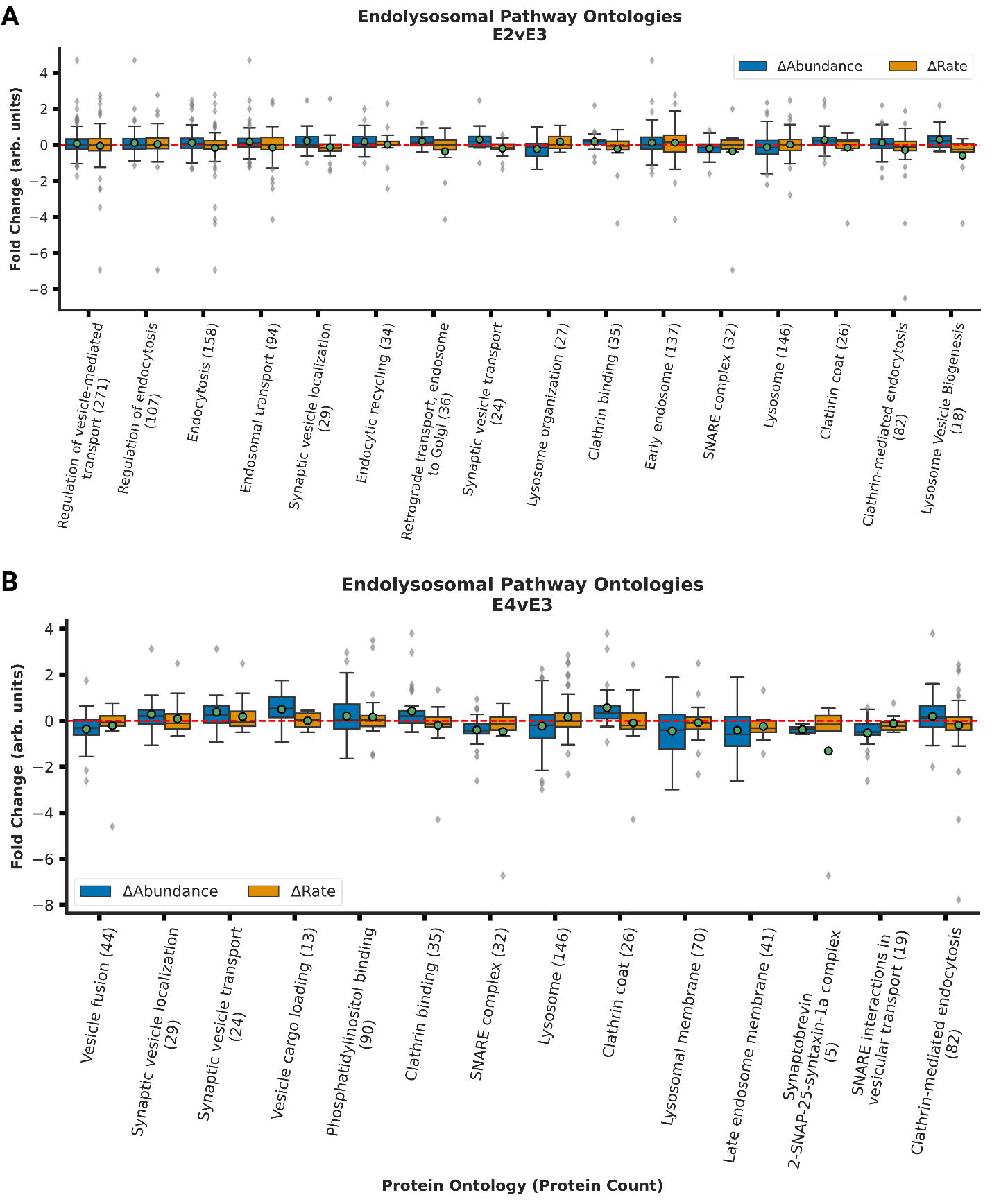


Fig C. Endolysosomal Ontologies

*Abundance and turnover FCs for ontologies related to endolysosomal processes in A) E2vsE3 and B) E4vsE3.*

**
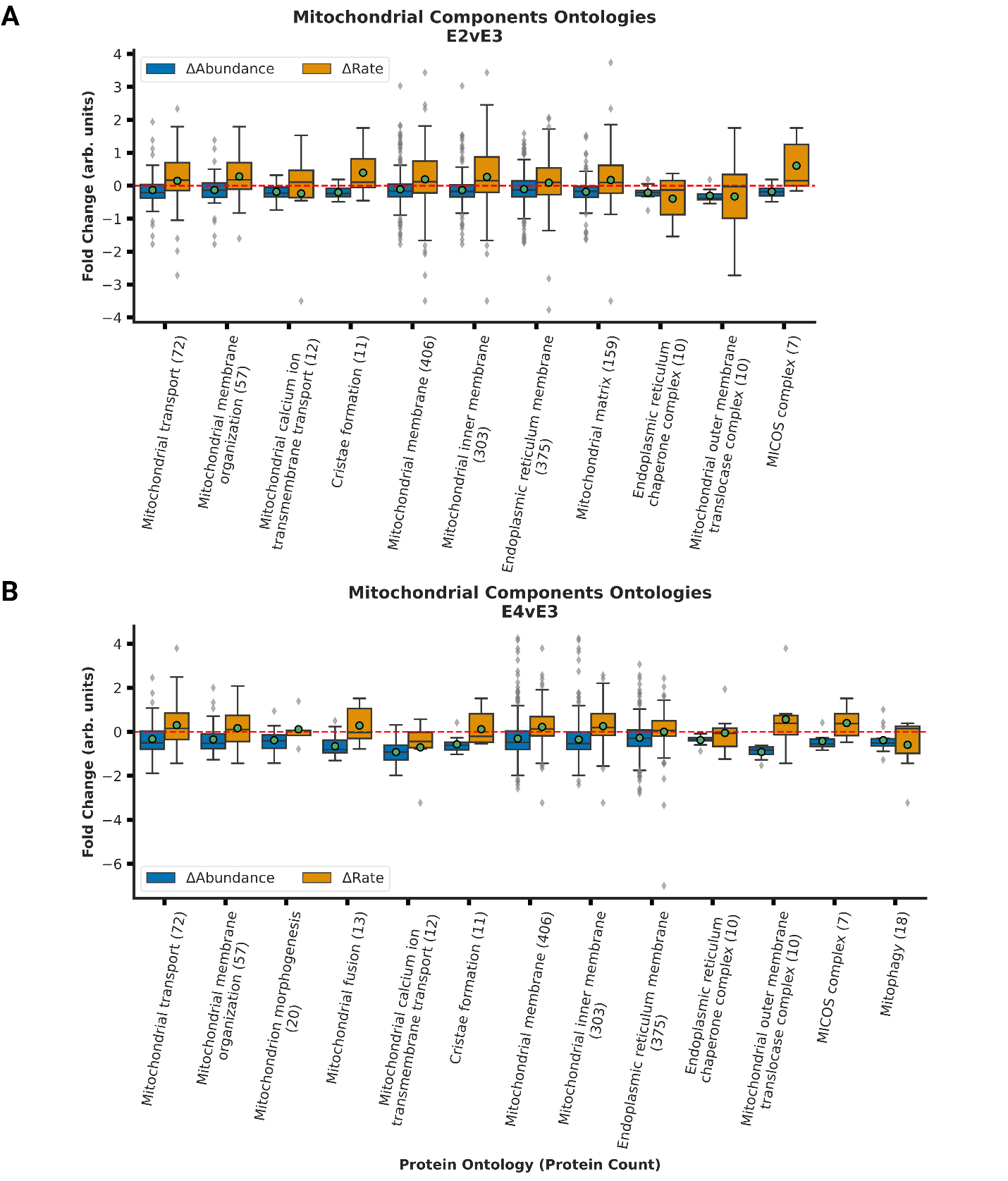
**

Fig D. Mitochondrial ontologies:

*Abundance and turnover FCs for ontologies related to mitochondrial components in A) E2vsE3 and B) E4vsE3.*


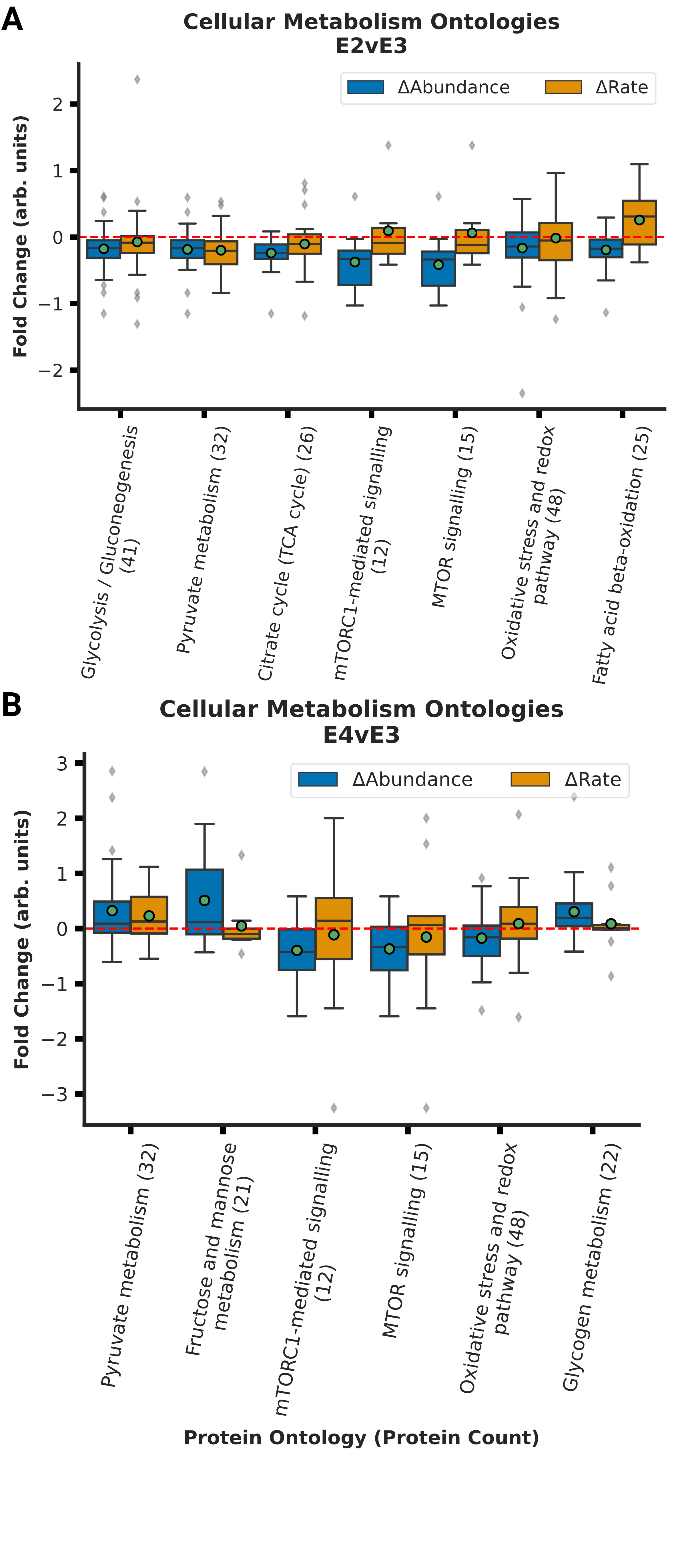


Fig E. Cellular Metabolism Ontologies

*Abundance and turnover FCs for ontologies related to cellular metabolism in A) E2vsE3 and B) E4vsE3.*


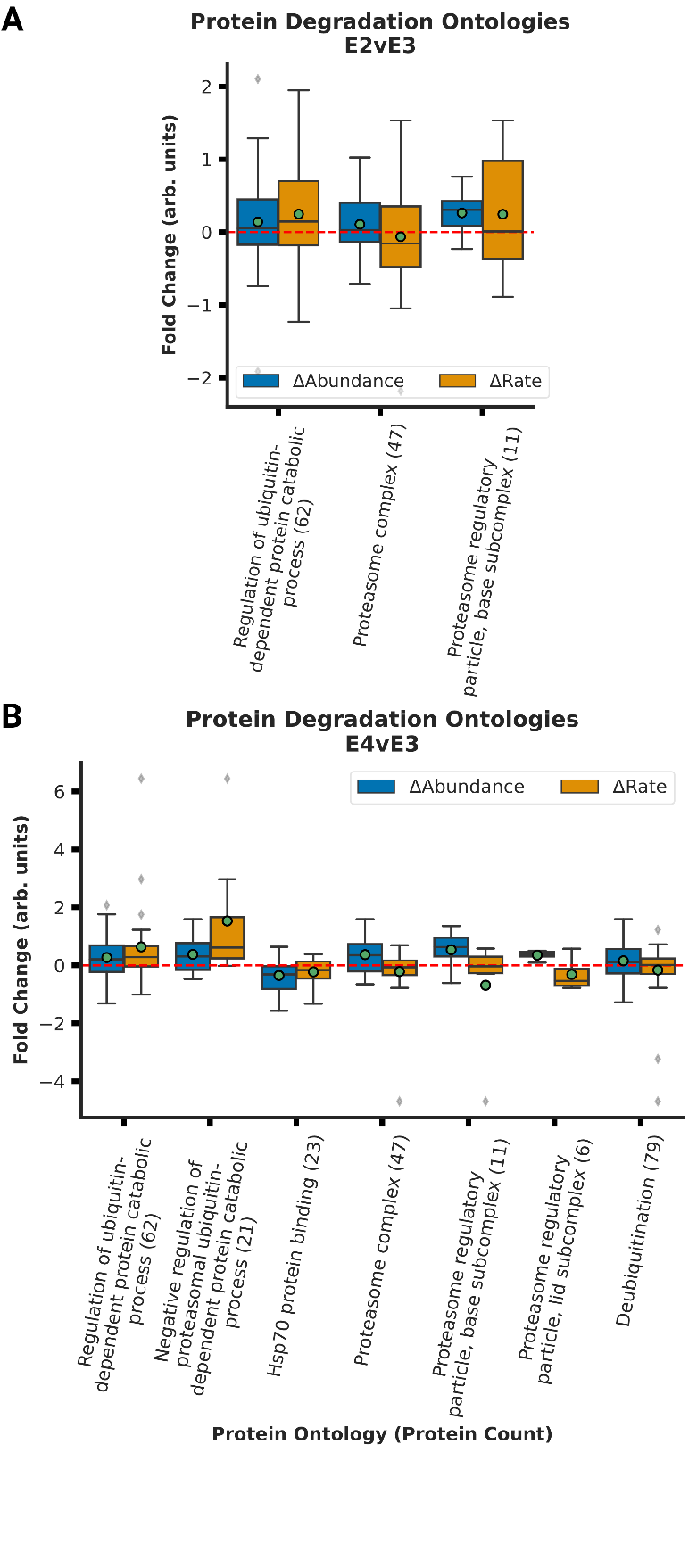


Fig F. Protein Degradation Ontologies

*Abundance and turnover FCs for ontologies related to protein degradation in A) E2vsE3 and B) E4vsE3.*

*
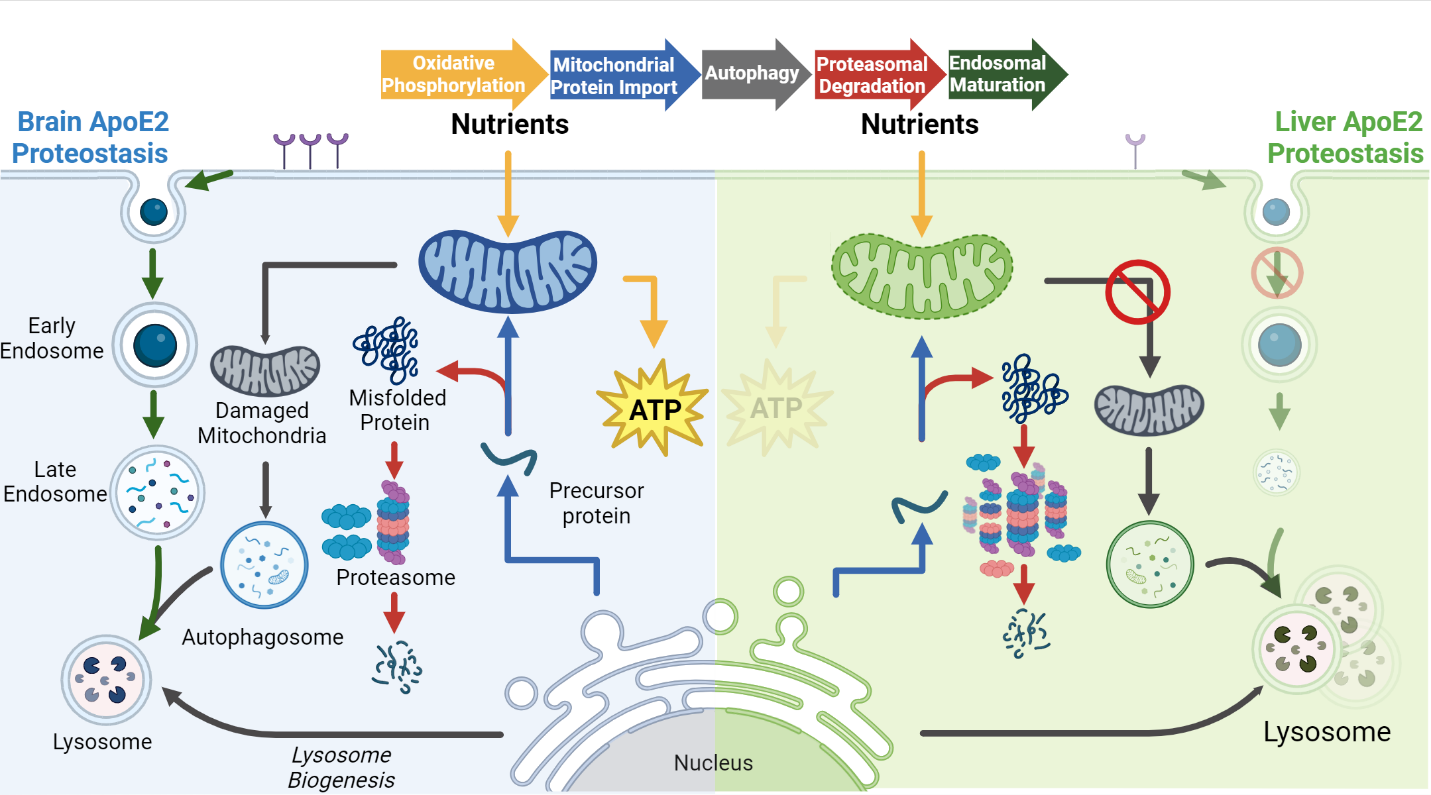
*

Fig G. ApoE2 change in brain (blue) versus liver (green) Model comparing the observed changes in proteostasis for ApoE2. The arrows are color coded to represent the different pathways impacted in both ApoE2 when compared to ApoE3


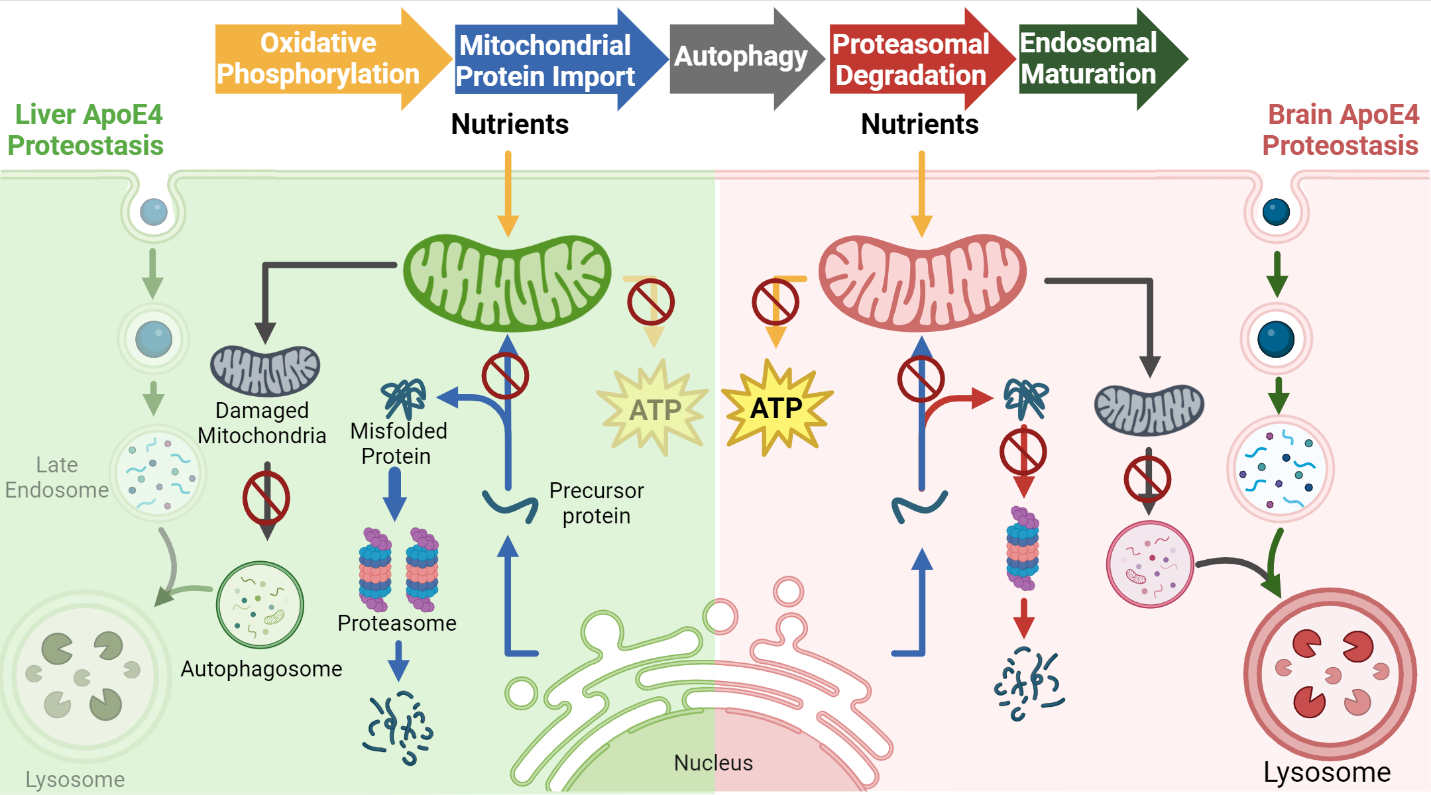


Fig H. ApoE4 change in brain (red) versus liver (green). The arrows are color coded to represent the different pathways impacted ApoE4 when compared to ApoE3
